# In(Ga)N Nanostructures and Devices Grown by Molecular Beam Epitaxy and Metal-Assisted Photochemical Etching

**DOI:** 10.3390/nano11010126

**Published:** 2021-01-07

**Authors:** Abdul Kareem K. Soopy, Zhaonan Li, Tianyi Tang, Jiaqian Sun, Bo Xu, Chao Zhao, Adel Najar

**Affiliations:** 1 Department of Physics, College of Science, United Arab Emirates University, Al Ain 15551, UAE; abdulkareemks@gmail.com; 2 Wuhan National Laboratory for Optoelectronics, School of Optical and Electronic Information, Huazhong University of Science and Technology, Wuhan 430074, China; lizhaonan@hust.edu.cn; 3 Key Laboratory of Semiconductor Materials Science, Institute of Semiconductors, Chinese Academy of Sciences & Beijing Key Laboratory of Low Dimensional Semiconductor Materials and Devices, Beijing 100083, China; tangtianyi@semi.ac.cn (T.T.); sunjiaqian@semi.ac.cn (J.S.); srex@semi.ac.cn (B.X.); 4 College of Materials Science and Opto-Electronic Technology, University of Chinese Academy of Science, Beijing 101804, China

**Keywords:** GaN, InGaN, photochemical etch, molecular beam epitaxy

## Abstract

This review summarizes the recent research on nitride nanostructures and their applications. We cover recent advances in the synthesis and growth of porous structures and low-dimensional nitride nanostructures via metal-assisted photochemical etching and molecular beam epitaxy. The growth of nitride materials on various substrates, which improves their crystal quality, doping efficiency, and flexibility of tuning performance, is discussed in detail. Furthermore, the recent development of In(Ga)N nanostructure applications (light-emitting diodes, lasers, and gas sensors) is presented. Finally, the challenges and directions in this field are addressed.

## 1. Introduction

Group III–V semiconductors, in particular, III-nitrides and their compounds, have recently received growing interest from the scientific community owing to their fascinating fundamental properties and novel applications. The outstanding properties of these materials, including wide direct bandgap, chemical and thermal stability, piezo/pyroelectricity, high conductivity/mobility, and biocompatibility, enable reliable operation of devices together with the awareness of different environmental and ecological challenges [1,2,3,4]. Nanostructured GaN and InGaN possessing the above properties have become revolutionary materials. The flexible bandgap range from 0.64 for InN to 3.45 eV for GaN, covering a uniquely ultra-wide spectrum [1], is ideal for broader applications. Advances in the synthesis of these materials have led to major developments in practical nanoscale smart objects, such as light-emitting diodes (LEDs), probes, photovoltaics, and lasers [5,6,7,8,9]. These materials are also used as efficient photoanodes in solar-powered water splitting, which turns an alternative energy source into fuel, albeit releasing CO_2_ in the process [10].

Nanostructures are usually prepared using bottom–up [11,12,13] or top–down [14] approaches. Well-aligned GaN and InGaN nanostructures have been obtained by metal–organic chemical vapor deposition (MOCVD) [15,16,17], molecular beam epitaxy growth (MBE) [18], the vapor–liquid–solid (VLS) mechanism [19], laser-assisted catalytic growth (LCG) [20], and the ion-etching reaction [21,22].

In this review, we focus on MBE and metal-assisted photochemical etching (MacEtch), which can be used to grow high-quality nitride nanostructures. MBE is a popular bottom–up method for growing arsenide materials and devices [23]. In recent years, MBE has also been widely used to grow nitride nanowires under nitrogen-rich conditions with a high V/III flux ratio, which promotes vertical growth and limits lateral growth [24]. The nanowire geometry confines the possible defects to the bottom of the structure, thereby ensuring high crystal quality in the upper active region and improving light extraction in LEDs. As the strain in nanowires (unlike that in nitride thin films) can be relaxed both out of plane and in-plane from the sidewall, the indium composition of InGaN nanowires can be substantially improved without worrying about increased strain, and the strain-related piezoelectric polarization field can be reduced. Furthermore, the structure can grow on different heterogeneous substrates, such as Si, metal, and quartz [24,25,26]. Due to their high quality and large specific surface areas, nitride nanowires have been adopted in LEDs, lasers, and water-splitting applications [27,28,29].

Both bottom–up growth and dry etching techniques are expensive and complex [12,30]. By contrast, wet etching can be accomplished via a more straightforward process at a lower cost with less material damage. The wet-etching process can be easily realized in a liquid reservoir such as a beaker, with the nanostructures obtained being intact without any surface lattice damage compared with those obtained using dry etching techniques. [31]. Several GaN and InGaN nanostructures have been fabricated by wet-etching methods, mainly via electrochemical and photochemical etching. Porous n-GaN can be synthesized via electrochemical etching in acidic (HF) or alkaline solutions [32,33,34] by applying an external current. The morphology of the etched material is influenced by the character and concentration of the electrolyte [35], material doping level, illuminance power, and sample bias [36]. A possible alternative to photoelectrochemical etching is metal-assisted chemical etching, which generates porous nanowire semiconductors using electroless techniques [37]. MacEtch is the most popular technique because it produces a homogeneous porous morphology over a wide region and is simpler than other surface-roughening techniques. [38,39]. MacEtch prevents the surface damage caused by dry etching procedures, which is desired for potential optoelectronic applications. MacEtch reactions can be broadly categorized into two types: single-step reactions with HAuCl_4_/HF [37], AgNO_3_/HF [40] or solutions containing AgNO_3_/HF/CH_4_O [38]; and two-step reactions involving predeposition of metal nanoparticles or patterned thin-film metals in the presence of HF/H_2_O_2_ [41].

## 2. Synthesis Methods for (In)GaN Nanostructures

### 2.1. GaN and InGaN: Porous and Nanowire Structures Prepared via MacEtch

MacEtch has become a well-developed technique for preparing Si nanostructures [42,43,44,45] and has recently been adopted for synthesizing GaN nanostructures. Najar et al. [46] synthesized GaN nanowires (NWs) using MacEtch. They coated 10-nm-thick strips of platinum separated by a few millimeters on a cleaned GaN film using a sputtering system and treated the films in a solution containing H_2_O_2_:HF:CH_3_OH (2:1:2) under ultraviolet (UV) light. Very long NWs (>10 μm) with a wurtzite structure were obtained. Geng et al. [38] investigated the growth of porous GaN in various metal catalysts and GaN doping environments and demonstrated an improvement in the MacEtch technique and the catalytic scheme in the order of Ag < Au < Ir < Pt. The authors also observed that the catalytic action of continuous films contrasted with that of films with discontinuous islands. Continuous metal films covered the surface tightly, obstructing the MacEtch reaction. This effect can be resolved by fabricating discontinuous films or increasing the intensity of laser irradiance. Doping enhanced the MacEtch activity in the order of p-GaN < intrinsic GaN < n-GaN. Benton et al. performed photoelectrochemical etching in KOH solution, creating nanoporous GaN and InGaN/GaN films with pore sizes varying between 25 and 60 nm [47].

Najar et al. [48,49,50,51] also fabricated porous GaN nanostructures by etching bulk GaN in a solution of H_2_O_2_, CH_3_OH, and HF with a volume ratio 2:1:2. They found a relationship between the elastic properties and porosity kinetics of the nanostructures; in particular, the elastic modulus of the structure decreased with a decrease in porosity.

**Figure 1:**
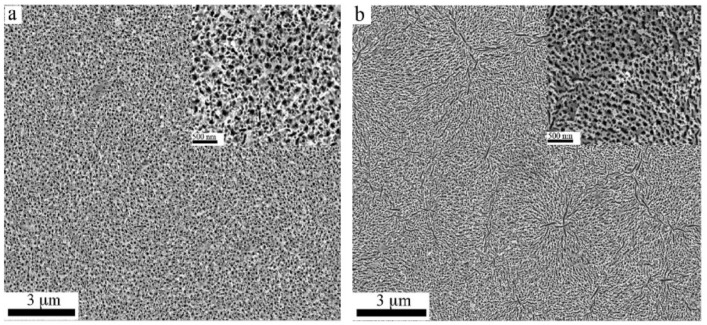
SEM of the porous GaN nanostructures prepared using electroless chemical etching technique: (**a**) for 45 min and (**b**) for 90 min. The insets are the zoom-in windows. Reproduced from [48], with permission from Copyright American Institute of Physics, 2012.

The etching time does not seem to affect the morphology and size of the resulting pores, but prolonging the etching duration increases the number of pores (see Figure 1). The approximate porosities of the samples etched for 45 and 90 min were 17.5% and 32%, respectively. The appearance of elongated pores in the 90-min etched sample was due to the fusion of cylindrical pores by the etchant, which narrowed the sidewalls between adjacent pores.

Perumal et al. [52] compared the porosifications of MacEtch-fabricated GaN in different porosification catalysts—hydrofluoric acid (HF) based (CH_3_OH:HF:H_2_O_2_), nitric acid (HNO_3_) based (CH_3_OH:HNO_3_:H_2_O_2_), sulfuric acid (H_2_SO_4_) based (CH_3_OH:H_2_SO_4_:H_2_O_2_), and platinum (Pt)—under UV enhancement. Under continuous UV illumination, the Pt catalyst increased the number of holes at the etchant–semiconductor interface by absorbing incident radiation, thereby increasing the etch rate. The authors formed a uniformly distributed network of nanopores with no ridge morphology. The pore size ranges of the HF, H_2_SO_4_, and HNO_3_-etched samples were 38–42, 42–47, and 14–18 nm, respectively (extended pores), and 18–20, 20–22, and 9–11 nm, respectively (small pores), with equivalent proportions of CH_3_OH and H_2_O_2_. The HNO_3_ etchant yielded smaller pores with excessive pore intensity, whereas the other etchants produced larger pores of similar size.

**Figure 2:**
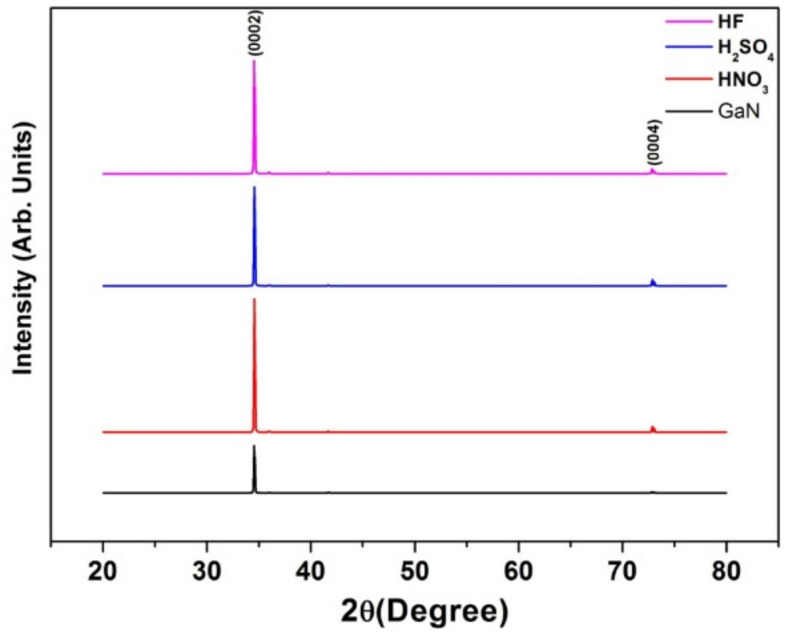
HRXRD patterns of the pristine sample and samples etched with different etchants. Reproduced from [52], with permission from Copyright World Scientific, 2016.

Figure 2 shows the X-ray diffraction (XRD) spectra of as-grown samples and samples obtained with various etchants. The higher crystallinity in the etched samples than in the as-grown samples, manifesting as high diffraction-peak intensity, was attributable to substrate-induced stress relaxation. The diffraction peaks at 34.59° and 76.35° are associated with the (0002) and (0004) GaN diffraction planes, respectively. Film formation was verified by the high intensity of the (0002) reflections along the *c*-plane. The GaN peaks of all etched samples confirmed that the GaN layer was not completely etched away and preserved its epitaxy. Zang et al. [53] produced single-crystalline, smooth-surfaced GaN nanowires (0002) via MacEtch over an electrodeposited metal catalyst (Au nanoparticles). The ideal moving path of the Au nanoparticles was along the (0002) face of GaN. GaN NWs can be many microns long, and their diameters range from 8 to 24 nm. The single peak at 34.5° in the XRD spectrum of the GaN nanowires was indexed to the (0002) wurtzite-type crystal face of GaN.

Wang et al. [54] studied the role of metal distribution in the formation of GaN nanowires and demonstrated that GaN effectively forms in AgNO_3_ solution or a mixed solution of CuSO_4_ and HF. They prepared metal-based etchants by combining concentrated AgNO_3_ or CuSO_4_ (0.01 M) with concentrated HF (5.0 M) and deionized water. The topology of the GaN surface changed significantly as the etching time increased from 10 to 50 min. The GaN film became porous after 10 min of etching under UV irradiation, and nanowires were created after 20 min of etching. Prolonging the etching time to 40 min increased the length of the nanowires.

**Figure 3:**
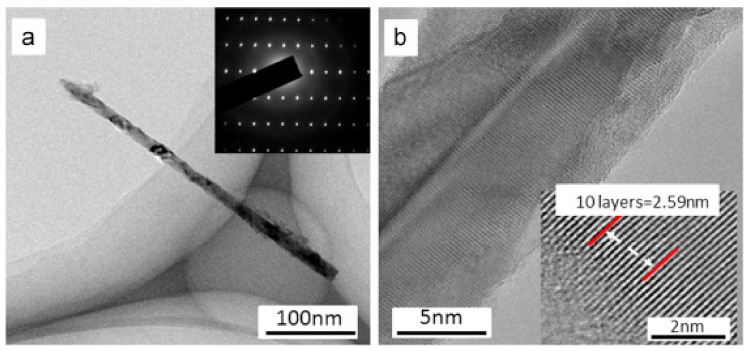
TEM images of nanowires on the GaN sample without metal coating and etched in AgNO_3_/HF for 50 min. (**a**) The image of a single nanowire. (**b**) The enlarged view of the nanowire in (**a**). Reproduced from [55], with permission from Copyright Elsevier, 2016.

Using MacEtch, Wang et al. [55] grew a single nanowire with a diameter of ≈25 nm and a rough surface, approaching the surface topography of Si NWs [56]. Figure 3a shows a transmission electron microscopy (TEM) image of their sample. The selected area electron diffraction pattern (Figure 3a, inset) confirmed the monocrystalline characteristics of the NWs. The enlarged image in Figure 3b clarifies the monocrystalline character of the NWs. The interplanar distance of the NWs was consistent with the *c*-plane lattice constant of GaN and verified the monocrystalline hexagonal structure of the NWs on the etched samples.

**Figure 4:**
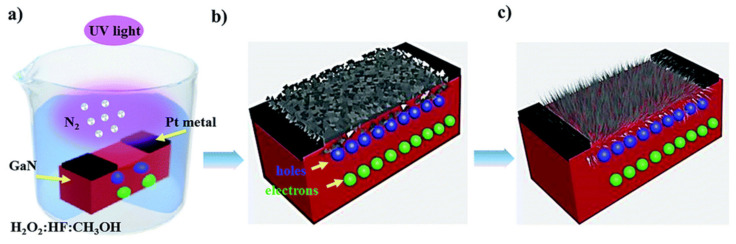
Schematic etching mechanism of GaN nanowires. (**a**) The reaction of the surface with H_2_O_2_: HF: CH_3_OH solution under UV light and generation of electron-hole pairs, (**b**) synthesis of porous GaN or InGaN for short etching times, and (**c**) synthesis of nanowires for longer etching times. Reproduced from [46], with permission from Copyright Royal Society of Chemistry, 2017.

Unlike the traditional anodic etching techniques for fabricating porous semiconductors, MacEtch is an electroless etching technique employing an unpatterned discontinuous metal layer in a solution containing HF and H_2_O_2_. MacEtch forms porous structures of Si and III–V semiconductor materials [57,58]. To initiate open-circuit local oxidation and reduction reactions, MacEtch utilizes noble metals. The process begins when the oxidant creates holes (h^+^) on the metal surface and then diffuses into the semiconductor. The holes are consumed by oxidizing the semiconductor directly below the metal, forming a soluble product in the acid solution [59]. The holes are generated via oxidant reduction catalyzed by cathode materials such as Au, Cu, Ag, and Pt deposited on the semiconductor surface. When these holes are introduced to the semiconductor valence band (VB), they oxidize and establish an ionic structure soluble in an acid solution (e.g., HF). By this process, the materials are eliminated despite the net metal utilization. The etching mechanism and chemical reactions during the MacEtch process are schematically shown in Figure 4. The formation mechanisms of GaN and InGaN nanostructures are similar and hence can be generalized.

GaN and InGaN nanostructures are formed via a three-phase process. First, under UV irradiation in the presence of CH_3_OH:H_2_O_2_:HF (2:1:2) etching solution, electron–hole (e–h) pairs are formed at the interface of GaN or InGaN–electrolyte. Aided by band bending at the substrate–electrolyte interface, the photogenerated holes oxidize the anode reaction at the GaN or InGaN surface while the electrons diffuse into the GaN or InGaN mass. The holes act as an anode, oxidizing the surface layer of GaN and InGaN with the evolution of N_2_ gas, forming Ga_2_O_3_ and In_2_O_3_, respectively.

**Figure 5:**
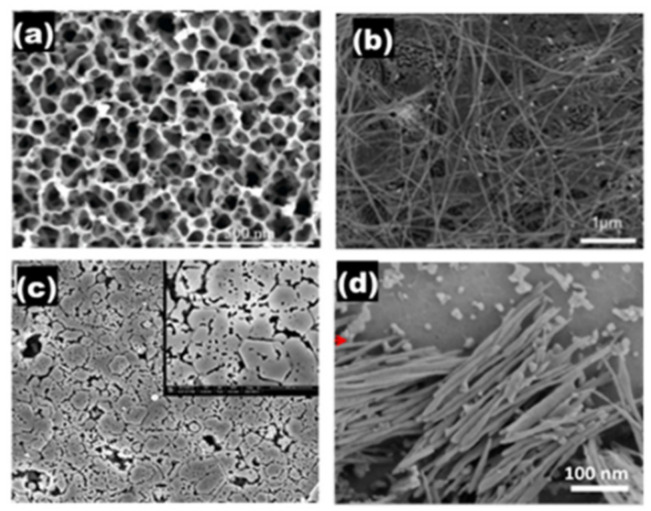
SEM images of (**a**) porous GaN, (**b**) GaN nanowires, (**c**) porous InGaN, and (**d**) InGaN nanowires. Reproduced from [38,51,60,61], with permission respectively from Copyrights IOP Publishing, 2013, The Optical Society (OSA), 2012, Elsevier, 2013, and Elsevier, 2017.

The overall etching duration plays a significant role in determining the morphology of GaN and InGaN nanostructures. In both cases, samples etched for a shorter period are porous, and further etching leads to nanowire formation. The etching of the GaN or InGaN layer leads to a porous structure resulting from epitaxial surface-layer dislocations and defects, followed by coalescing of the surface and vertical progression along the crystalline structure. After a sufficiently long etching time, the surfaces of GaN and InGaN are fully covered with vertical GaN and InGaN nanowires. As the etching time is further increased, the density of the nanowires becomes high, as seen in Figure 5b,d.

**Figure 6:**
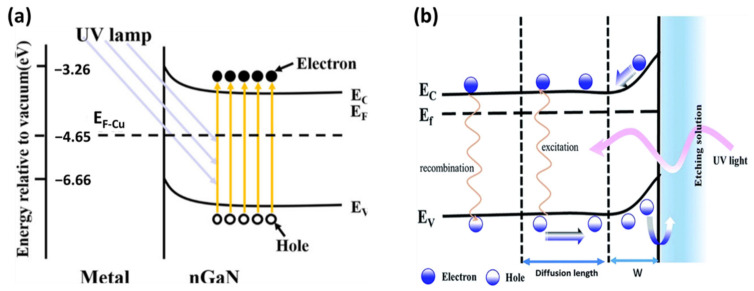
Energy band diagram of (**a**) GaN and (**b**) InGaN. Reproduced from [54] and [46] ((**a**) and (**b**) respectively), with permission from Copyrights Elsevier, 2019, and Royal Society of Chemistry, 2017.

The etching mechanism, which occurs by oxidation and subsequent dissolution of the semiconductor surface, relies mainly on the photogenerated e–h pairs. Holes play a significant role in increasing the oxidation state of the surface atoms. The number of surface holes for the oxidation reaction is increased by the absorption of incident radiation. The etching process forms bubbles through the evolution of nitrogen. The etching time relies on the involvement of the surface charge region at the n-GaN–electrolyte boundary, which results from the Fermi level equilibrium between the GaN surface and the electrochemical potential of the electrolytes (see Figure 6).

The metal-assisted photochemical electroless etching of n-GaN starts when electron-hole pairs are excited by the incident UV photons. The photo-generated holes enter the interface of the GaN/electrolyte and are located at the surface bonds, allocating oxidation to the surface gallium atoms. The holes oxidize the surface layer to generate Ga_2_O_3_ gas via the evolution of N_2_, as seen in the following Equation (1) [62], and function as an anode.

2GaN + 6h^+^ + 3H_2_O → Ga_2_O_3_ + 6H^+^ + N_2_ (1)

The reduction reaction of H_2_O_2_ by Equation (2) occurs in platinum as the cathode for water generation.

H_2_O_2_ + 2H^+^ → 2H_2_O + 2h^+^ (2)

HF’s reaction to Ga_2_O_3_ is the second step. This reaction occurs on the surface of GaN crystal grains, on the boundaries of the GaN crystallite grain, and on the dislocations of the surface [63]. The Ga_2_O_3_ oxide dissolution occurs to form GaF_3_, as shown in Equation (3).

Ga_2_O_3_ + 6HF → 2GaF_3_ + 3H_2_O (3)

The dissolution of Ga_2_O_3_ oxide in the HF solution will improve the baffling bonds and be responsible for the positive oxide charges under insufficient oxygen conditions [64]. Repulsion from the positive oxide surface of H^+^ charged ions in the solution will reduce the dissolution rate of Ga_2_O_3_ by Equation (4) on the surface of the crystal grain.

Ga_2_O_3_ + 6H^+^ → 2Ga^3+^ + 3H_2_O (4)

However, dislocated sites and defects are negatively charged [64,65], and dissolution of Ga_2_O_3_ can be effectively commenced at surface defect sites at a much higher rate owing to HF [66]. Pores are formed on the GaN surface for shorter etching periods. The final phase is for longer etching times as the pores are etched in the GaN layer across the vertical (0001) crystallographic direction at the dislocation sites, leaving behind NWs structures. The GaN Nanowires then get longer and collapse after prolonging a couple of microns [54].

Depending on the local current flux and the complete disposal of the oxidized semiconductor required to maintain the local electrochemical reaction), MacEtch can produce both solid and porous nanostructures. The porosity is controlled by varying the oxidant/acid ratio in the solution and the type and design of the catalyst. The various synthesis parameters for the development of GaN and InGaN porous nanowires are given in Table 1 and Table 2, respectively. MacEtch reactions occur only at the metal–semiconductor interface in controlled etching environments. Consequently, the metal sinks into the semiconductor while the semiconductor is etched just below the metal, serving as a mask to resist etching. When the metal catalyst is shaped in any manner and length, the pattern gravitates into the semiconductor, creating microstructures and nanostructures such as pillar arrays and reverse hole structures [37]. GaN nanostructures ranging from dendritic extensions to highly monodisperse collections of GaN nanowires can be prepared by varying the solution composition, concentration, and etching time under UV illumination [40]. GaN has a more favorable V_B_ (–6.66 eV relative to vacuum) than metals such as Au, Ag, Cu, and Pt. The UV illumination assists in hole formation in the VB during the GaN etching process. The electrons in the V_B_ of GaN gain sufficient energy to excite the conduction band under UV illumination, creating holes in the V_B_ simultaneously.

**Figure 7:**
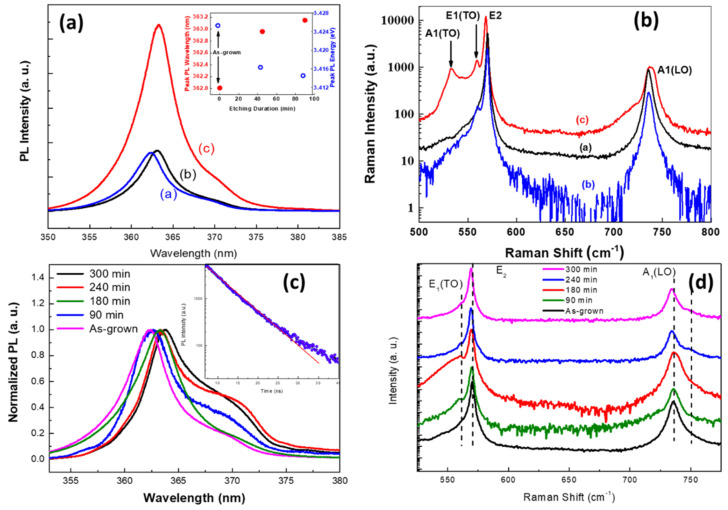
PL and Raman spectra of porous nanowires of GaN. We reproduced (**a**,**b**) from [48], with permission from Copyrights American Institute of Physics, 2012. And reproduced (**c**,**d**) from [46], with permission from Royal Society of Chemistry, 2017.

Najar et al. observed a redshift in the peak position of the photoluminescence (PL) spectrum of porous GaN samples [48] as shown in Figure 7a, which contrasts with the blue shift reported in porous Si nanostructures caused by quantum size effects [79]. The relief in the porous GaN samples can be related to uniaxial stress. The scattering of light from the pores’ sidewalls improves the photon removal effectiveness and decreases the dislocation density in the GaN porous structures; accordingly, the PL intensity increases with an increase in porosity. Benton et al. [47] noted a similar redshift of the emission wavelength in GaN samples. In their study, they found that the GaN epitaxial layer experienced significant compressive strain, causing a 16% lattice mismatch between GaN and its sapphire substrate. The strain could be relaxed to a large extent because of the development of the nanoporous structures, leading to a redshift in the PL peak position that depended on the extent of relaxation. Perumal et al. [52] reported a similar redshift in the near-band-edge emission peaks of their etched porous samples, which appeared at ≈363.5 nm. Evidently, the defect density in etched samples is higher than that in as-grown samples because the etching process increases the specific surface area and removes some of the nanostructures. Raman spectroscopy provides information on vibrational states that are sensitive to crystallinity, stress, carrier concentration, and mobility. Therefore, Raman spectra can be used to monitor the formation process and growth of GaN nanowires (see Figure 7b). Group theory suggests four active Raman modes in GaN nanowires, which normally crystallize into a hexagonal wurtzite structure: *E*_2_^L^, *E*_2_^H^, *E*_1_(TO), and *A*_1_(LO) [80]. Typically, the peaks of these four modes appear around 143, 658, 556, and 724 cm^−1^, respectively. Xi et al. performed a Raman study of GaN nanowires produced on anode aluminum oxide templates [81]. They allocated the Raman peaks at 143, 556, 568, and 733 cm^−1^ to the *E*_2_^L^, *E*_1_(TO), *E*_2_^H,^ and *A*_1_(LO) modes of first-order phonons, respectively. A peak at 752 cm^−1^ emerged with a reduction in the GaN NW diameter. An acoustic phono-overtone was attached to the 315 and 418 cm^−1^ peaks. Under an incident laser, the high specific surface area of the NWs resulted in additional molecular vibrations that dramatically increased the strengths of the *E*_2_^L^, *E*_2_^H^, *A*_1_(LO), and *E*_1_(TO) peaks of GaN NWs from those of planar GaN. For GaN nanowires with a diameter of 60 nm, a different Raman surface mode (Frohlich mode) at ≈815 cm^−1^ was observed. Najar et al. observed a new Frohlich mode peak at 750.5 cm^−1^ in anisotropically etched GaN NWs (see Figure 7d), which they attributed to the anisotropy [46]. They noted a lower frequency shift ascribable to the stress relief that occurred during longer etching periods.

### 2.2. Exfoliation of Porous Structure and NWs

**Figure 8:**
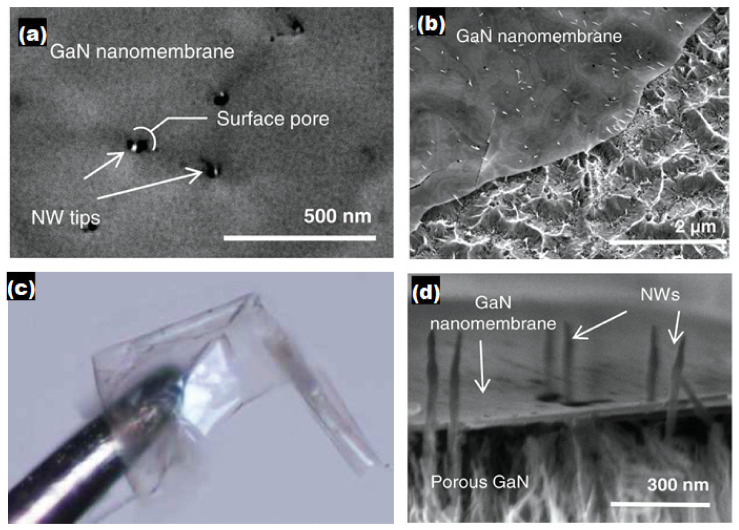
(**a**) SEM image showing the tips of the NWs below the surface pores. (**b**) Inclined top–view SEM image of the nanomembrane on top of the porous GaN. (**c**) The exfoliated GaN nanomembrane held at the tip of a tungsten probe. (**d**) Inclined cross-sectional SEM image of NWs protruding through the surface pores in the GaN nanomembrane. Reproduced from [98], with permission from Copyright WILEY-VCH Verlag GmbH & Co. KGaA, 2013.

GaN membranes have been prepared via diverse processes: laser [82], chemical [83], electrochemical [84,85], and mechanical lift-off from suitable buffer layers [86,87]. However, the direct growth of inorganic NWs on flexible substrates is hampered by interfacial reactions between the grown nanowires and flexible metallic substrates and the low melting temperatures of flexible organic substrates [88]. Doan et al. lifted an InGaN/GaN LED from a sapphire surface using a 248-nm KrF excimer laser [89]. Chung et al. deposited a GaN LED directly on a thin-film layer of ZnO-coated graphene and mechanically lifted it from the substrate [90]. Subsequently, Kobayashi et al. mechanically lifted a GaN LED from a boron nitride buffer layer [91]. Cheng and co-workers demonstrated transferable substrate-less GaN LED grown on SiC buffer layer [92]. Voronenkov et al. removed the GaN layer by laser cutting [93]. The lifted GaN was transferred onto a copper substrate. Despite these advances, existing methods involve several steps and often require costly equipment. These limitations have impeded the further production of GaN crystal membranes. Xiao et al. synthesized self-standing nanoporous GaN membranes via electrochemical reaction and found the nanopore morphology is related to the n-doping concentration [94]. Yoo et al. [95] fabricated a GaN LED on a polyamide substrate using a standard soft lithography technique. The flexible GaN LED was mechanically and optically stable on a flexible substrate, surviving a bending test of radius up to 3.5 mm and a cycling test up to 2000 times. GaN can be transferred from sapphire to a flexible substrate by laser lift-off and contact printing [96]. A flexible GaN membrane platform on a silicon wafer developed by eutectic bonding and a laser lift-off process offers flexibility for the stable and reliable design of various pixel shapes and related manufacturing processes. Flexible GaN has also been developed using other techniques such as the selective anisotropic etching of N-polar GaN in hot phosphoric acid [97]. However, there are few reports on the preparation of flexible GaN via electrochemical etching methods. ElAfandy et al. [98] realized threading dislocation-free (TD-free) nano-membrane generated from MacEtch of GaN template-substrates, as shown in Figure 8. The threading dislocations were carefully separated during the etching process, forming nanowires, while the ~25 nm thick nanomembrane was remaining unetched on the surface of the substrate. The GaN nanomembrane, the NWs, and the porous area are visible clearly in Figure 8a,b. The microscopic picture (Figure 8c) clearly shows its flexibility. Ultralong GaN NWs were obtained as shown in Figure 8d if the etching process persists for a prolonged time.

### 2.3. Nitride NWs Grown by MBE

Under high-vacuum conditions, atoms of one or more types constituting an epitaxial film are sprayed as atomic beams onto the substrate. Some of the bombarded atoms form an ordered crystal film on the substrate by undergoing a physical–chemical process. The In, Ga, and Al beams (group III) are generated by thermal evaporation, whereas reactive N beams are formed by NH_3_ or N_2_ plasma, which can also be used to grow III-nitrides (important for growing In-nitride) at low temperatures. The high-vacuum chamber not only provides a long mean-free-path for the beam atoms but also promotes the development of high-quality (In, Al)GaN nanostructures without any catalyst. The growth parameters can be accurately controlled via in situ monitoring capability. Although the growth rate in MBE is low, it has proven very successful in the growth of optoelectronic device structures [99,100].

Specific substrates are required for epitaxial growth. Homoepitaxial growth is favored because it avoids thermal and lattice mismatches, which degrade the quality of the epitaxial layer. This is important for the epitaxial growth of optoelectronic device structures based on GaAs. However, in the epitaxial growth of III-nitrides, this approach is rendered difficult by the small size and high price of the GaN or AlN substrates. At present, sapphire, silicon carbide, and silicon substrates are the primary substrates for epitaxial GaN. Table 3 gives the material parameters of the III-nitride epitaxy. The lattice parameters of conventional substrates largely differ from those of GaN, leading to an elevated density of dislocations in epitaxial GaN films.

The high dislocation density has restricted the growth of GaN film on the abovementioned substrates. However, researchers have successfully grown GaN nanowires on these substrates [107]. In nanowire epitaxy, the dislocation density is reduced by the effective strain relaxation [108,109] and low polarization fields [110].

**Figure 9:**
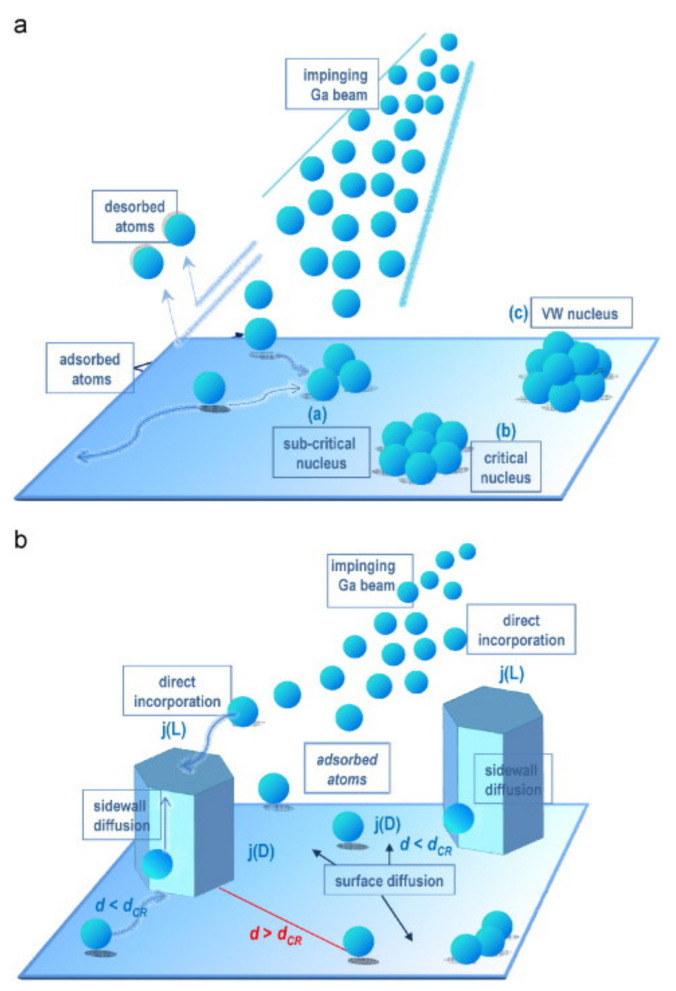
The schematic of (**a**) the nucleation process of nanowires in Volmer–Weber (VW) growth mode. (**b**) Nanowire growth from stable nuclei. Reproduced from [112], with permission from Copyright Elsevier, 2008.

Nanowires grow by a sequential process of absorption, desorption, nucleation, and growth, as shown in Figure 9. GaN nuclei have different diameters and their locations and nucleation times are random, resulting in high dispersal. Nanowire growth is driven by Ga diffusion along the sidewalls, and the growth rate is inversely proportional to the diameter of the NWs. When the nucleus reaches a critical size, its subsequent growth is attributed to the direct incorporation of Ga atoms that hit the apex of the NWs and diffuse to the bottom of the NWs. After reaching the substrate, they climb up to the apex along the sidewall [111,112].

Using the MBE process, Yoshizawa et al. [113] grew self-organized GaN nanopillar structures on a (0001) sapphire substrate in a radio-frequency (RF)-generated radical source. The obtained nanopillars were dense along the *c*-axis and remained perpendicular to the growth plane. By studying the effect of the growth conditions on the nanopillars, they found that the growth rate, density, and diameter of the nanopillars increased with an increase in the N_2_ flow rate, and the nanopillar diameter could be increased by increasing the RF power. Therefore, the nanopillar sizes can be regulated by controlling the N_2_ gas flow rate and RF power [113]. Araki et al. observed that when GaN is grown on a sapphire substrate by electron cyclotron resonance MBE, H_2_ supports the epitaxial layer and stimulates the three-dimensional columnar growth of GaN [114]. Namkoong et al. [115] investigated the effect of nitridation temperature of the sapphire substrate on subsequent growth. Prior to the growth of GaN on sapphire, the substrate was exposed to an N_2_ plasma atmosphere for nitridation at 200 °C in plasma-assisted MBE. A uniform Al–N layer singularity was formed on the surface of the nitrided sapphire substrate, providing a nucleation point for the subsequent GaN growth at 500 °C. The nitridation significantly improved the crystal quality of the following layer [115].

**Figure 10:**
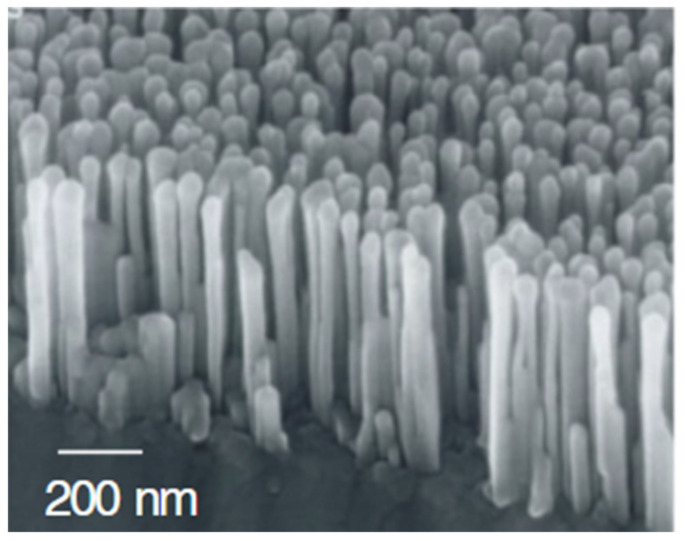
SEM image of GaN nanopillars on sapphire. Reproduced from [116]. with permission from Copyright WILEY-VCH Verlag GmbH & Co. KGaA, 2004.

Kikuchi et al. performed high-temperature nucleation of Al–N and self-organized growth of GaN nanopillars on a (0001) sapphire substrate after low-temperature nitridation in RF-MBE. They controlled the diameter, height, and density of the nanopillars by changing the growth conditions. The final density, average diameter, and height of the nanopillars were 2 × 10^10^ cm^−2^, 80 nm, and 1.8 μm, respectively. Figure 10 shows a scanning electron microscopy image of the nanopillars. The strong emission peaks in the PL spectrum of the GaN nanopillars confirmed their high crystal quality, which is essential for optoelectronic applications [116]. Ramesh et al. grew a vertically distributed GaN nanowall network (NWN) by applying laser-assisted MBE technology on a nitrided sapphire substrate. The high crystal quality of the GaN NWN was demonstrated by the small full-width-at-half-maximum (FWHM) in the XRD rocking curve test. Furthermore, they found that the nitridation temperature of the substrate influences the type of stress developed in the epitaxial GaN layer [117].

GaN nanostructures have also been grown on silicon substrates, which are economical and can be produced in large quantities. Kikuchi et al. developed GaN nanopillars on Si (111) substrates. Unlike the direct nitridation of sapphire substrates in an N_2_ plasma atmosphere, silicon substrates should be processed under a Ga beam before passing into N_2_ plasma to form GaN dots, which serve as the initial nucleation sites for subsequent growth. They developed GaN nanopillars with a diameter of 80–100 nm, a height of 1500-nm, and a density of 1.2 × 10^10^ cm^−2^. The threshold optical power density of stimulated emission from the obtained GaN nanopillars was as low as 300 kW/cm^2^ [118].

Bertness et al. produced GaN nanowires on Si (111) without metal catalysts. The nanowires were well separated, and their diameters and lengths were around 50–250 and 2–7 μm, respectively. The authors suggested that growth via this mechanism (unlike catalytic growth) depends partly on the stability of the nonpolar GaN planes [119]. Meijers and colleagues [120] studied the relationship between the morphological/optical characteristics and growth parameters of GaN NWs grown via MBE on Si (111) substrates. They found that by optimizing the equivalent pressure of the Ga beam ramp, tapering and agglomeration could be prevented. The growth was driven by self-catalysis of the Ga droplets.

**Figure 11:**
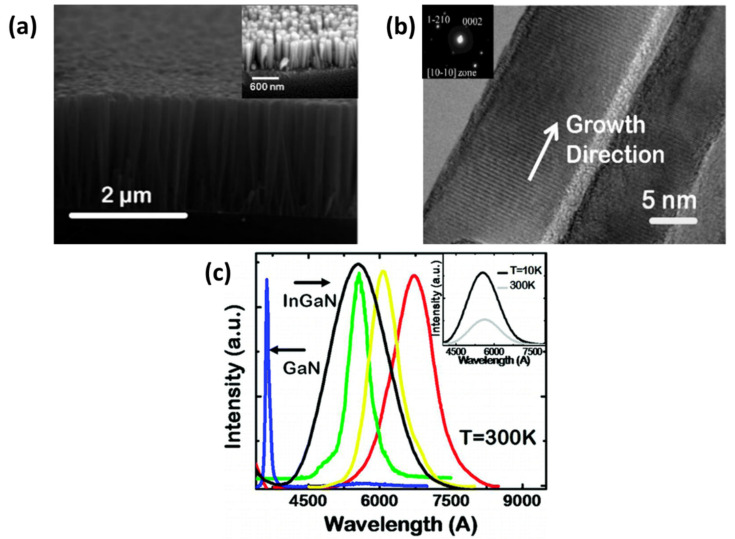
(**a**) Cross-sectional SEM image of InGaN nanowires grown on Si. (**b**) HRTEM image of InGaN nanowire. The inset shows the selective area diffraction pattern of the nanowire. (**c**) Room-temperature PL spectra of InGaN nanowires with different In compositions. The inset shows the 10K and RT PL of nanowires. Reproduced from [121], with permission from Copyright American Chemical Society, 2010.

Guo et al. demonstrated the catalyst-free production of InGaN NWs of diameter ranging from 10 to 50 nm with high density (1–2 × 10^11^ cm^−2^) on a (001) Si substrate, as shown in Figure 11. The NWs were crystallized into a wurtzite structure with relatively few defects compared to nitride films. The emission of the NWs could not only be tailored from UV to red but could be broadened to a white emission by changing the composition. The intrinsic quantum efficiency varied from 20% to 35%. The radiative and nonradiative lifetimes of the carriers were 5.4 and 1.4 ns, respectively. Both green- and white-emitting planar LEDs were also produced, with a relatively low quantum-confined Stark effect and bandtail filling effects [121].

Guo et al. developed GaN NWs on a silicon (111) substrate using plasma-assisted MBE, and also grew InGaN NWs at low temperatures to prevent precipitation of In. TEM images showed that the NW diameters were uniformly distributed in the radial direction, with very few dislocations; consequently, the GaN NWs delivered an excellent performance. The authors regulated the composition of the InGaN layer by changing the growth conditions during the epitaxy process, thereby changing the NW emission spectrum. In the time-resolved PL spectrum of the nanowires, the radiative recombination lifetime was higher than the nonradiative recombination lifetime, further confirming the low defect density in the NWs [121].

Mi et al. grew AlN and AlGaN NW in an RF-MBE system. The proper size and density of the AlN nanowires were achieved by controlling the GaN nanowire seed layer. As revealed in the PL emission spectrum, the properties of the AlN nanowires largely depended on the growth temperature of the initial GaN nanowires. Mg-doped AlN nanowires achieved high-efficiency p-type conduction owing to their high crystal quality. Meanwhile, the PL spectrum of the AlGaN nanowires revealed the high application potential of Al(Ga)N nanowires in UV and deep-UV luminescence [122].

Kruse et al. performed selective-area MBE growth of GaN NWs on SiO_2_-masked Si (111) substrates. The NWs were directly grown on the Si substrate, and an oxide mask was formed with no additional buffer or mask materials. When the diameter of the mask window was less than 50 nm, most of the NWs were straight and vertical. The diffusion length was also influenced by the window spacing; when the spacing exceeded 1 μm, the ratio of window space to unoccupied window space was almost unrelated to the window density [123].

**Figure 12:**
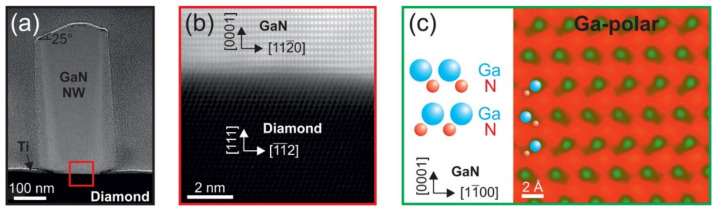
(**a**) STEM image of a GaN NW grown on the diamond substrate. (**b**) The heterointerface between diamond and GaN. (**c**) High-resolution STEM image of the GaN nanowire. Reproduced from [124], with permission from Copyright American Chemical Society, 2015.

NWs have also been developed on high-thermal-conductivity substrates. Schuster et al. developed GaN NWs and nanotubes on a diamond substrate via MBE supported by a structured Ti mask (see Figure 12). The Ga-polarity of the wurtzite NWs suppressed the parasitic growth at high substrate temperatures (890 °C), made the heterointerface more abrupt, and improved its quality [124].

Zhao et al. fabricated excellent InGaN/GaN NWs as quantum-disk LEDs on various metallic substrates using MBE. Mo substrates with high electrical and thermal conductivity can realize high-power light emitters; moreover, an integrated n-contact surface and reflector (typically used for LEDs) can form additional TiN layers on the Mo substrate. A sacrificial Ti intermediate layer is appropriate for LED-epitaxy-lift-off, and the Ti interlayer on Mo can be sacrificed to realize reusable substrate technologies [24].

The lattice mismatch between GaN and SiC is 3%, which is much smaller than that between GaN and silicon or sapphire substrates. SiC is an ideal substrate for forming high-quality GaN nanowires, but its high cost and small size have limited its wider application. Recently, some researchers have tried to grow a SiC buffer layer on a silicon substrate and then transfer the substrate to the MBE process for growing GaN NWs. Similar to the method for growth on the silicon substrate, after a thermal cleaning process, the SiC/Si (111) was exposed to a Ga beam at low temperature for a while to form nano-islands, which served as nucleation points for subsequent GaN NWs. The GaN NWs obtained on SiC/Si exhibited a significantly higher PL intensity than those grown directly on silicon, demonstrating their high crystal quality [125].

MBE growth of III-nitride nanowires requires no catalysts, and the self-organized growth mode dramatically improves the density and uniformity of the NWs. The large specific surface area of the NWs formed by MBE greatly reduces the defect density in the crystals. For this reason, MBE-formed nitride NWs have excellent applications in optoelectronic fields, such as LEDs and lasers.

## 3. Applications of (In)GaN Nanostructures

### 3.1. Application to Gas Sensors

The capability to identify, rapidly detect, and monitor different gases at low concentrations is highly desired in medical, industrial, security, and even domestic applications while working in a dynamic mode. For example, H_2_, H_2_S, and C_2_H_4_ are common pollutant gases that pose a serious risk to human health [126,127]. This scenario requires unified gas sensors with excellent selectivity, reliability [128], high sensitivity [129], fast response, long-term stability, cost-effectiveness, and workability at low gas concentrations [130,131,132,133]. An ample gas-sensing span is also necessary [134]; the ideal range of sensing gas detectors is 10–1000 ppm [135]. Gas detectors that can maneuver in highly corrosive environments and at extreme temperatures are mandatory in the automotive and aerospace industries [134,136,137].

**Figure 13:**
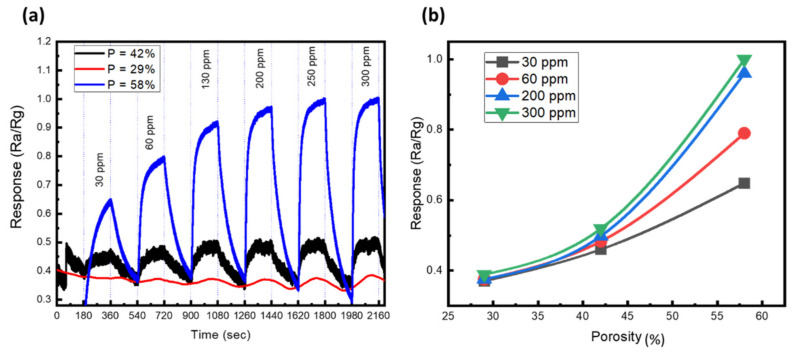
Effect of porosity on H_2_ detection at room temperature: (**a**) versus time and (**b**) versus the porosity. Reproduced from [138], with permission from Copyright American Chemical Society, 2019.

Shafa et al. fabricated a sensor from sulfur-treated platinum-coated porous GaN, which detected 25 ppm of H_2_ gas at ambient temperature as shown in Figure 13 [138]. They tested the performance of the H_2_ gas sensor over a range of operating temperatures, fixing the gas concentration at 30 ppm and supplying an adsorption activation energy of 22 meV. The results demonstrated the high capacity of the porous GaN sensor. Additionally, the detection response of the sulfur-treated porous GaN gas sensor improved after increasing the porosity and pore size (see Figure 13b). The authors then developed a concentration model of H_2_ gas based on the surface reaction equation (Knudsen diffusion) and equipped it within the porous GaN. The model revealed that small pores restrict the diffusion of hydrogen.

**Figure 14:**
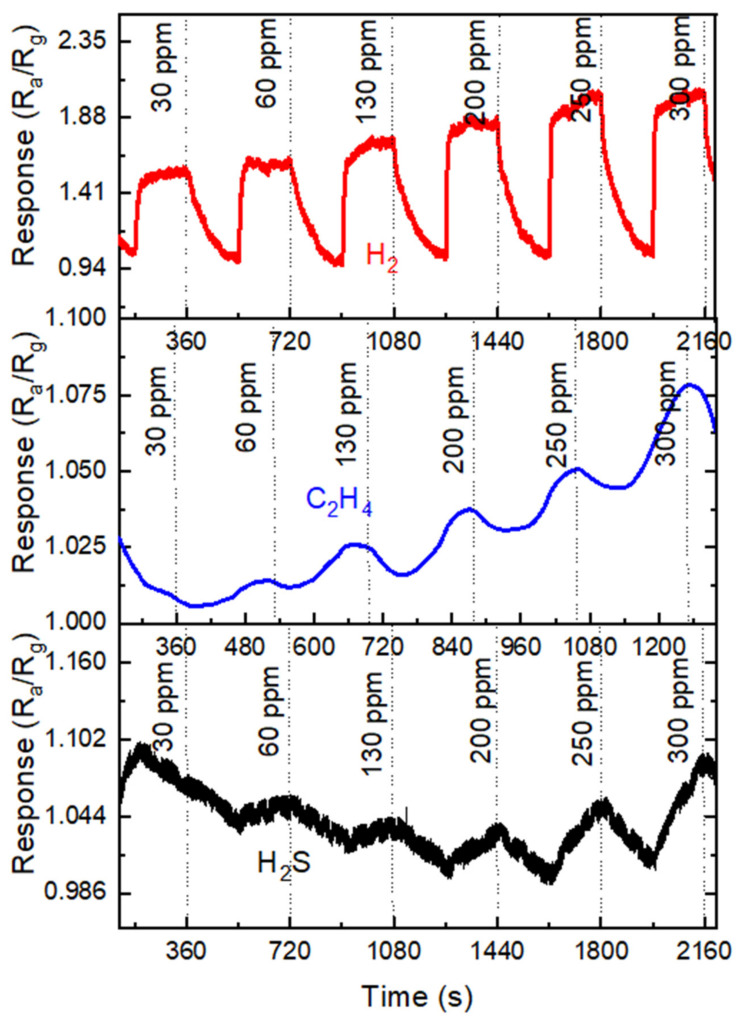
Sensor responses for H_2_, C_2_H_4_, and H_2_S gases at 23 °C. Reproduced from [139], with permission Copyright Elsevier, 2020.

Figure 14 shows the room-temperature responses of the sensor to three gases: H_2_, H_2_S, and C_2_H_4_. The response to H_2_ rose steeply as the H_2_ concentration increased from 30 to 300 ppm. The response to H_2_ at 30 ppm was the strongest, and the response to C_2_H_4_ was second-highest because although the active region of the sensor was passivated, the sensor’s response to C_2_H_4_ gas was nonuniform and independent of the gas concentration. However, the sensor’s response to H_2_S gas was lower, and the behavior was repeatable. The low response to H_2_S was ascribed to the filling of the porous surface with adsorbed H_2_S atoms, which might cease additional adsorption and interplay of the gas molecules in the active region.

### 3.2. Application of Nitride Nanostructures in LEDs

With their low defect density, enhanced light extraction, and low polarization field, LED structures based on III-nitride nanowires can potentially realize next-generation solid-state illumination, full-color displays, and communication through visible light.

**Figure 15:**
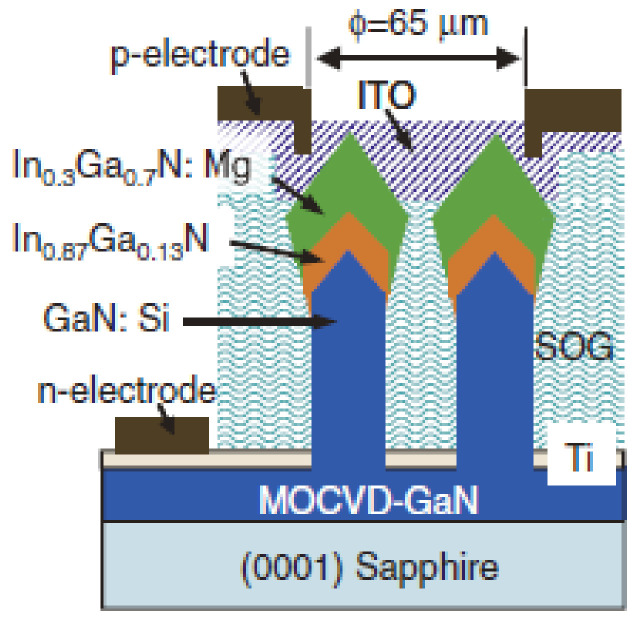
Schematic of the LED structure on the sapphire. Reproduced from [140], with permission from Copyright The Japan Society of Applied Physics, 2012.

Applying the RF-MBE technique, Kishino et al. grew a triangular lattice array of n-type doped GaN nanopillars on a sapphire substrate. They fabricated InGaN nanopillars with high In composition and p-type doped InGaN nanopillars with low In composition (see Figure 15). At ambient temperature, the turn-on voltage of the fabricated LED unit was 1.0 V. Electroluminescence spectroscopy demonstrated an emission wavelength of 1.46 μm from the LED unit. This work revealed the advancement possibilities of InGaN nanostructures in infrared LEDs and detectors [140].

To control the emission spectrum of InGaN quantum dots, Nguyen et al. modified the active- region composition of the quantum dots by adjusting the growth conditions of the MBE system. Finally, they fabricated InGaN/GaN dots-in-the-GaN wire LEDs on a silicon (111) substrate. The PL spectrum of the dots-in-the-wire structure was adjustable over a broad range of visible-light wavelengths. When InGaN dots with different In compositions were grown in the same NW, intense white-light emission with a peak wavelength at 545 nm and a wide FWHM in the PL spectrum was observed. Moreover, the TEM image confirmed that the quantum dots in the NWs had different In compositions, which were responsible for the broad white-light emission. The internal quantum efficiency and current density of their LED device reached 20% (at room temperature) and 200 A/cm^2^, respectively [141]. This InGaN quantum dot structure, with its three-dimensional quantum confinement and adjustable In composition as the active region, covered the entire visible-light range in the GaN NWs, which is ideal for LED lighting and display applications.

Philip et al. fabricated full-color emission LED devices on a silicon substrate by controlling the In composition of the InGaN/AlGaN quantum dot active region in GaN NWs. The active region between a Si-doped GaN layer and an Mg-doped GaN layer contains 10 layers of InGaN/AlGaN quantum dots. Depending on the In composition, the emission wavelength of the fabricated LED varied between 460 and 600 nm. Within the unique InGaN/AlGaN core–shell structure, the carriers were confined to the InGaN quantum dots, reducing the surface nonradiative recombination of the carriers and significantly improving the efficiency of carrier injection. It is worth noting that by growing InGaN quantum dots with different compositions in AlGaN NWs, Philip et al. also achieved strong white-light emission. This tunable NW LED is applicable to advanced solid-state lighting [142].

**Figure 16:**
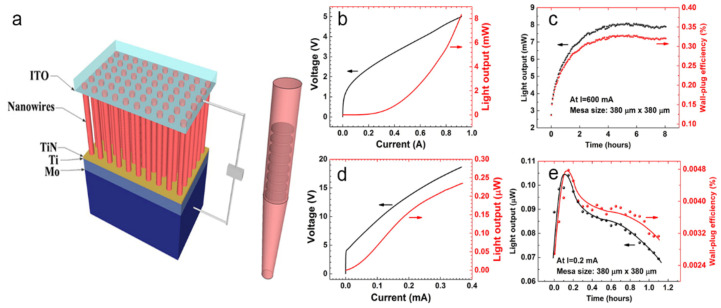
(**a**) Schematic of the LED structure grown on Mo substrate. (**b**) and (**d**) Light output power−current−voltage (L–I–V) curves of LEDs on Mo substrate and Si substrate, respectively. (**c**) and (**e**) Light output power and wall-plug efficiency of LEDs on Mo substrate and Si substrate, respectively. Reproduced from [143], with permission from Copyright American Chemical Society, 2016.

Zhao et al. realized high-power, reliable InGaN/GaN quantum disk-in-the-wire LEDs with an emission wavelength of 710 nm on a Mo substrate. Before growing the NWs, they deposited a 500-nm-thick Ti layer on the substrate. The LED structure contained n-type GaN and p-type GaN for the electrode contacts, and the active area included eight layers of InGaN quantum disks separated by GaN barrier layers (see Figure 16a). They compared the performances of the LED devices formed on the Mo and silicon substrates. Owing to the excellent thermal conductivity of Mo, the wall-plug efficiency and output power of the LED were significantly higher on the Mo substrate than on the silicon substrate. The authors performed a soft burn-in test of an InGaN/GaN LED device of size (380 × 380) μm^2^ on the Mo substrate at 600 mA. In this test, the device ran continuously for at least 8 h, which was much higher than the running time of LEDs on a silicon substrate (1.1 h). The remarkable lifetime of the fabricated NW LEDs was attributed to the excellent heat-dissipating role of the Mo substrate. The high-power, high-reliability NW LEDs studied in this work were very suitable for high temperatures, harsh environments, and visible-light communication applications [143].

The unique characteristics of NWs make them more promising for LED lighting and display applications than conventional (In)GaN films. In particular, nanowire components can be controlled by changing the growth conditions, realizing a wide range of emission wavelengths from ultraviolet to infrared.

### 3.3. Application of Nitride Nanostructure in Lasers

Lasers based on silicon technology, especially electrically pumped lasers, have been a long-term objective of scientists and technologists. The most challenging part of integrating silicon photonics is finding or developing a suitable monolithic silicon-based laser. Electrically injected lasers can be realized via three common routes based on Si technology [144]: wafer bonding of IIIpi epitaxy laser structures on a Si wafer, the use of lasers with a built-in tensile strain and degenerate doping, and the direct growth of III–V quantum wells or quantum dots on misoriented silicon substrates (offcut (001) Si). In the first method, the IIIpi substrates limit the size of the bonding area. The second method forms Ge-on-Si p–n–n heterojunction diodes that allow direct radiative recombination in the indirect bandgap of Ge. The third approach is incompatible with COMS devices.

By virtue of their wide bandgap range, suitable surface-recombination velocity, and large exciton binding energies (typically 60 meV for AlN and 26 meV for GaN), the III-nitrides are potent competitors in the laser field [145]. In recent years, three-dimensional (3D) III-nitride quantum-confined nanowire lasers have attracted wide interest as alternatives to traditional planar lasers, which always possess high threshold current densities (several kA/cm^2^ for GaN-based devices).

**Figure 17:**
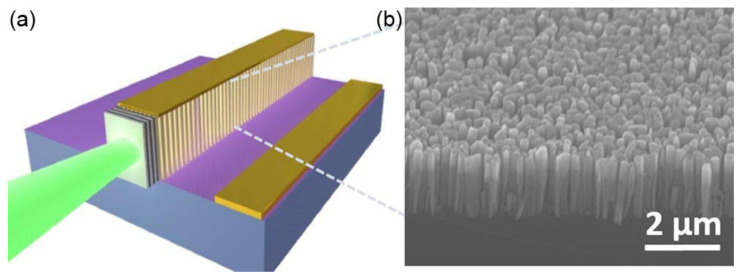
(**a**) Schematic of the nanowire laser structure. (**b**) SEM image of the disk-in-nanowire laser. Reproduced from [144], with permission from Copyright American Chemical Society, 2014.

In 2014, Frost et al. demonstrated the first electrically pumped laser edge-emitting disk-in-nanowire array of InGaN/GaN, which emits at 533 nm on a (001) Si substrate (see Figure 17). The array was fabricated via plasma-assisted MBE [144]. Thanks to a thin SiN_x_ buffer layer formed at the heterointerface of Si–GaN, and large surface-to-volume ratio, III-nitride nanowires formed directly on Si exhibited a lower defect density than III–V grown on typical lattice-mismatched substrates [146]. Moreover, this high-performance array was built on cheap Si substrates, which improved not only its performance but also cost-effectiveness. The threshold of Frost et al.’s disk-in-nanowire laser was 1.72 kA/cm^2^, and the lifetime was ≈7000 h. The multiple InGaN disks embedded in the GaN layers served as the gain medium, and therefore, it was possible to tune the emission wavelength by changing the composition. Hazari et al. grew In_0.85_Ga_0.15_N disks separated by In_0.4_Ga_0.6_N barriers as the active region and fabricated a 1.2-μm infrared laser with a threshold current density *J*_th_ and *T*_0_ of 1.24 kA/cm^2^ and 242 K, respectively [147]. In the same year, Jahangir et al. proved that by modifying the In component during the growing process of InGaN disk-in-nanowires could behave as quantum dots in terms of electrical characteristics [148].

**Figure 18:**
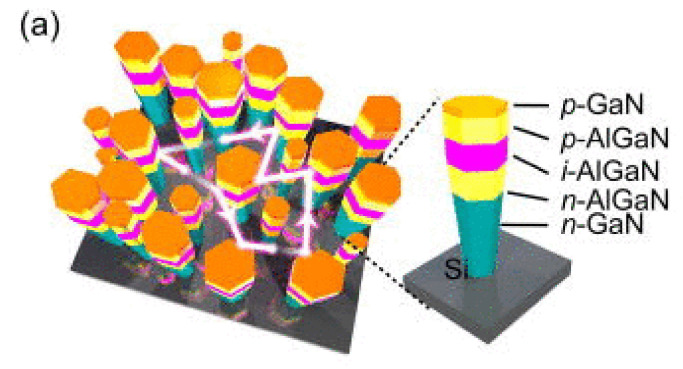

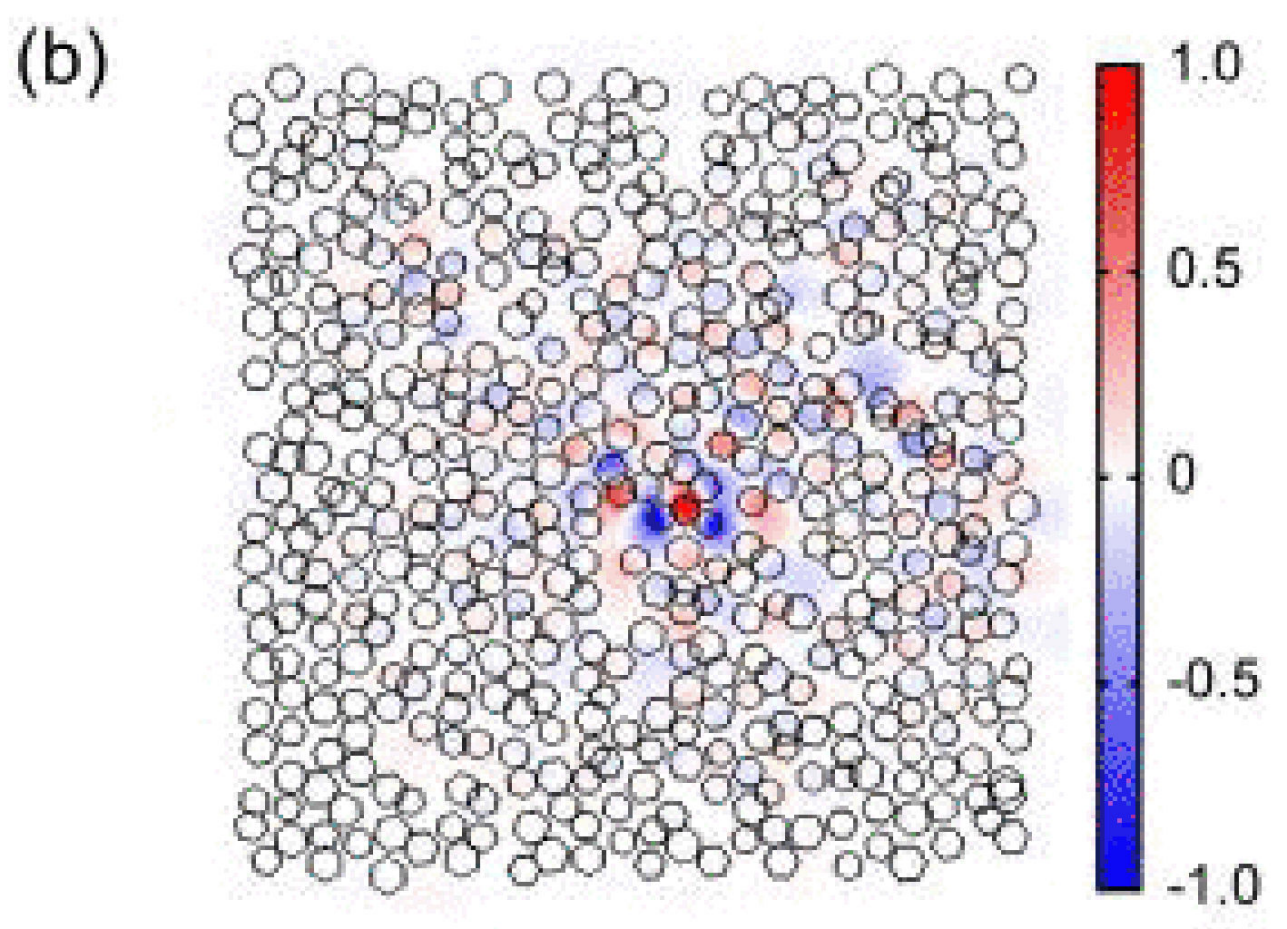
(**a**) Schematic of a random cavity inside AlGaN nanowire arrays; (**b**) simulation result of the electric field for a high-Q cavity formed by AlGaN nanowires. Reproduced from [150], with permission from Copyright American Institute of Physics, 2016.

Moreover, it is worth mentioning that AlGaN-based lasers can potentially cover a wide range of ultraviolet wavelengths, as the AlGaN bandgap ranges from 3.4 to 6.2 eV. Nevertheless, the wide bandgap and large carrier effective mass limit the densities of the carrier to the order of 10^19^ cm^−3^, meaning that the measured transparency current density *J*_tr_ is around 30 kA/cm^2^. Therefore, this family of materials is of limited applicability in practice. After multiple quantum-confined nanostructures, density-of-states adjustment, and reduction of defect densities and dislocations, the thresholds can be drastically reduced and the gain can be boosted. For instance, Zhao et al. reported an electrically pumped deep UV (≈289 nm) laser [149]. They formed nanoclusters functioning like quantum dots/dashes by modifying the Al incorporation in AlGaN nanowires. Besides the gain media, high-quality optical cavities are essential in lasing devices. Li et al. [150] carefully designed an AlGaN core–shell NW double-heterostructure array, which met the requirement of the Anderson localization of light and eventually achieved a UV-AII band laser (≈320–340 nm). Shortly afterwards, Zhao et al. shortened the lasing wavelength to UV-C (≈262.1 nm) at an operating temperature of only 77 K. Finally, their group achieved a high-performance electrically pumped deep UV (≈239 nm) laser that operates at room temperature [151,152]. All of these lasers incorporated QD-like nanostructures within perfectly grown AlGaN NWs and possessed high-Q cavities utilizing Anderson localization, as shown in Figure 18.

## 4. Challenges and Future Directions

Most semiconductors are formed by isotropic wet etching in the depth and lateral directions, which prevents the creation of high aspect ratio characteristics. Meanwhile, dry etching can be steered as the etchant accelerates toward the surface and is ionized in the gas phase. MacEtch, however, produces microstructures and nanostructures with anisotropic high aspect ratios on semiconductors. As a wet-etching method without requiring high-energy ions, MacEtch helps to alleviate the lattice damage caused by the dry etching process. MacEtch also uses no harmful gases and overcomes the limitation of etching depth when features have limited lateral dimensions [153]. Straight pores with a high aspect ratio cannot be achieved using traditional wet-etching techniques, but MacEtch can reasonably control the diameter and shape of the pores and can be applied in a broad, parallel process, which is easier to implement than highly energetic methods [37]. Moreover, MacEtch reduces the roughness of the sidewall compared with that of RIE [31]. Elaborate preparations such as contacting and adjusting voltages are needed in anodic wet etching but are avoided in MacEtch because deep pores are electrochemically etched by biasing the substrate [154].

In summary, MacEtch is a simple, feasible, and effective wet-etching technique for efficiently fabricating semiconductor nanostructures with high aspect ratios. MacEtch-based approaches have produced ordered GaN and InGaN NWs in chemical hoods without the use of expensive lithography instruments. From the abundant reports in this area, we can understand the mechanism and behaviors of etching. However, the mechanism underlying the etching direction remains to be explored. In MacEtch, controlling the direction of etching remains a challenge. InGaN nanostructures manufactured through MacEtch are relatively scarce in the scientific community. More development in MacEtch controllability can be expected; for instance, MacEtch can be combined with traditional electrochemical etching [155] or applied to the fabrication of III-nitride, III–V, and II–VI semiconductors. Many applications, including solar cells, gas sensing, water splitting, LEDs, and other optoelectronics whose structures are presently developed by bottom–up or dry etching processes, would greatly benefit from this simple manufacturing method. Meanwhile, MacEtch is expected to realize planar structures with high aspect ratios, but this realization demands advancements in surface passivation, uniformity, and controllability. We believe that MacEtch technology will be able to replace conventional surface-texture techniques such as alkaline and acid etching in the future.

(In)GaN nanowires grown via MBE have shown great potential in LEDs, lasers, and various devices. However, owing to the nature of nucleation, the nanowires are often uniform in diameter, length, and distribution. Nanowire devices are high-quality products but much more complicated to fabricate than conventional thin-film devices. The nanowire growth must be controlled from the nucleation stage by some procedure, such as patterning the substrates. A 3D fabrication process will help in ensuring compatibility with standard devices, which will avoid the planarization of the nanowires.

The transfer of devices to flexible substrates for bendable devices or to a heat sink for heat dissipation is limited by low-scale yield. The direct growths of NWs on flexible substrates and heat sinks have been reported by different groups but only at a small scale. Moreover, laser structures grown on heat sinks face huge problems during laser fabrication. A high-quality transfer process for applications such as displays and laser sources is urgently needed.

NWs grown via the MBE process have advantages such as high crystal quality, high p-type doping efficiency, and low-temperature growth. The combination of MOCVD and MBE will lead to high-performance nitride devices, leverage the high growth rate and quality of nitride by MOCVD, and exploit the abovementioned advantages of nitride nanostructures grown via MBE. Increased uptake of products for industrial applications can be expected.

## 5. Conclusions

GaN and InGaN nanostructures (porous and nanowires) have attracted significant attention because they are easy to be controlled during fabrication using MacEtch or MBE methods. The fabricated nanostructures present enhancements of their optical and electrical properties compared to bulk material, which will improve the device’s properties. In addition, the used techniques (MacEtch or MBE methods) present a huge potential for practical applications in light, lasers, gas sensors, and even wearable devices by transferring the material on flexible substrates. Based on these various fabrication processes, significant progress has been made towards applications in optoelectronic devices; however, several challenges are still exciting.

## Figures and Tables

**Table 1:** Parameters for the formation of porous GaN and GaN nanowires via metal electroless etching.

	GaN Substrate	Etching Solution	Etching Time (min)	Parameters of the Structures	Ref.
Porous GaN	MOCVD-grown, Si-doped, carrier conc. 1.2 × 10^18^ cm^−3^	CH_3_OH:HF:H_2_O_2_ (1:4:1)	60–120	D:55–60 nm	[67]
	MBE-grown on Si, carrier conc. 4 × 10^19^ cm^−3^	HF:CH_3_OH:H_2_O_2_ (2:1:1)	10–25–35	D:80–110 nm	[68]
	HVPE on sapphire, n = 3 × 10^16^ cm^−3^	CH_3_OH:HF: H_2_O_2_ (1:2:1)	60–120		[69]
	MBE-grown on Si, carrier conc. 4 × 10^18^ cm^−3^	HF:CH_3_OH:H_2_O_2_ (4:1:1)	10–25–35	D:80–110 nm	[70]
	-	CH_3_OH:HF: H_2_O_2_ (1:2:2)	60	D:80–100 nm	[71]
	Si-doped grew on sapphire, electron conc. 1–3 × 10^18^ cm^−3^	CH_3_OH:HF: H_2_O_2_ (1:4:1)	-	-	[72]
	Si-doped, HVPE on sapphire, conc. 1–3 × 10^18^ cm^−3^	CH_3_OH:HF: H_2_O_2_ (1:2:2)	60		[73]
	MOCVD grown n-GaN on SiC,	CH_3_OH:HF: H_2_O_2_ (1:1:1)	15–40		[58]
	MOCVD grown n-GaN on Al_2_O_3_, n = 1 × 10^17^ cm^−3^	HF:CH_3_OH:H_2_O_2_ (1:4:4)	15–60	-	[74]
	n-GaN grown on sapphire, conc. 3.4 × 10^17^ cm^−3^	HF:CH_3_OH:H_2_O_2_ (2:1:2)	45–90	≈65 nm	[48]
	GaN grew by HVPE, conc. 1 × 10^17^ cm^−3^	CH_3_OH:HF: H_2_O_2_ (1:2:2)	15–75	0.5 μm depth	[75]
GaN Nanowires	Si-doped, HVPE on sapphire, con: 4 × 10^18^ cm^−3^	HF: H_2_O_2_ (4M:1M)	50	8–24 nm	[53]
	MOCVD on sapphire, conc. 1 × 10^17^ cm^−3^	HF:CH_3_OH	10–50	200–300 nm	[55]
	MOCVD on sapphire, conc. 1 × 10^17^ cm^−3^	HF:H_2_O (1:1)	30–90		[76]
	HVPE on sapphire, conc. ≈10^18^ cm^−3^	AgNO_3_:HF: H_2_O/CH_3_OH	5–30	D:15–25 nm	[40]
	HVPE on sapphire, con: 4.8 × 10^18^ cm^−3^	K_2_S_2_O_8_:KOH (0.1M:1M)	30	D:13–52, 8–50 nm	[77]
	MOCVD grown GaN, conc. 3 × 10^18^ cm^−3^	AgNO_3_:HF:DI&CuSO_4_:HF:DI	10–50	1.8 μm depth	[54]
	Si doped, n-type GaN, resistivity 0.05 Ω.cm	H_2_O_2_:HF: CH_3_OH (2:1:2)	180	D:35 nm	[78]
	Si doped, n-type GaN, resistivity 0.05 Ω.cm	H_2_O_2_:HF: CH_3_OH (2:1:2)	90–300	D:35 nm	[46]
	HVPE grown n-type GaN, conc. ≈10^18^ cm^−3^	CH_3_OH: H_2_O_2_:HF (1:2:2)	30	D:50–100 nm	[39]

**Table 2:** Parameters of InGaN nanowire formation by metal electroless etching.

InGaN Substrate	Etching Solution	Etching Time (min)	Parameters of the Structures	Ref.
Undoped InGaN grew on doped GaN, conc. 5 × 10^17^ cm^−3^	CH_3_OH:H_2_O_2_:HF(2:1:2)	10–30	280 nm length Nanowires	[61]
Undoped InGaN grown on Si-doped n-type GaN, resistivity 0.05 Ω·cm	CH_3_OH:H_2_O_2_:HF (2:1:2)	30	280 nm length Nanowires	[78]

**Table 3:** Basic epitaxy parameters of III-nitrides and conventional substrates.

Material	Structure	Lattice Constant (Å)		Thermal Expansion (10^−6^ K^−1^)		Thermal Conductivity (W/cm·K)	Ref.
		a	c	a	c		
GaN	Wurtzite	3.189	5.186	5.59	3.17	1.3	[101]
AlN	Wurtzite	3.11	4.979	5.27	4.15	2.85	[102]
InN	Wurtzite	3.533	5.693	3.8	2.9	0.45	[103]
Sapphire	Wurtzite	4.765	12.982	5.0	9.03	0.23	[104]
SiC (6H)	Wurtzite	3.0730	15.118	4.3	4.7	4.9	[105]
Si	Diamond	5.431		2.6		1.3	[106]

## Data Availability

Not applicable.
